# Bordetella hinzii Antenatal Sepsis in a Pregnant Woman With Sickle Cell Disease and Intrauterine Fetal Demise: A rare and first case report from India

**DOI:** 10.7759/cureus.113601

**Published:** 2026-07-29

**Authors:** Monika Kulkarni, Pooja Shendre, Vaishali Taywade, Sangeeta Bhalavi, Sunanda Shrikhande

**Affiliations:** 1 Microbiology, NKP Salve Institute of Medical Sciences and Lata Mangeshkar Hospital, Nagpur, IND; 2 Microbiology, All India Institute of Medical Sciences, Nagpur, IND; 3 Microbiology, Government Medical College and Hospital, Nagpur, IND

**Keywords:** antenatal sepsis, blood culture, bordetella hinzii, fetal demise, maternal sickle cell anaemia

## Abstract

*Bordetella hinzii* is a rare, opportunistic Gram-negative bacterium traditionally associated with poultry but increasingly identified as a human pathogen in immunocompromised individuals. We report the first Indian case of *B. hinzii* causing antenatal sepsis in a 27-year-old pregnant woman with homozygous sickle cell anaemia and β-thalassemia minor, leading to intrauterine fetal demise at 24 weeks of gestation.

The patient presented with breathlessness, edema, and absent fetal movements. Laboratory results showed severe anaemia, leucocytosis, and raised inflammatory markers. Blood cultures yielded *B. hinzii*, confirmed by the VITEK 2 system (bioMérieux, Marcy-l'Étoile, France). The isolate was susceptible to meropenem, piperacillin/tazobactam, cefepime, ceftazidime, and co-trimoxazole, but resistant to aminoglycosides and ceftriaxone. The patient improved with empirical therapy, though fetal loss had occurred.

This case highlights the emerging clinical significance of *B. hinzii* in high-risk pregnancies and the need for advanced microbiological tools and antimicrobial therapy in managing rare opportunistic infections.

## Introduction

Bordetella hinzii is a Gram-negative coccobacillus that has recently emerged as a rare but significant opportunistic pathogen in humans, especially among high-risk and immunocompromised individuals. The genus Bordetella comprises several species classified into ‘classical’ - B. bronchiseptica, B. pertussis, and B. parapertussis - and ‘non-classical’ species including B. hinzii, B. holmesii, B. petrii, B. avium, B. trematum, ‘B. ansorpii’, B. sputigena, B. bronchialis, B. flabilis, B. muralis, B. tumbae, and B. tumulicola [1].

Vandamme et al. first isolated and described B. hinzii from poultry in 1995. Since then its zoonotic potential and human pathogenicity have been acknowledged [2]. Bacteremia [3], pneumonia [4,5], urinary tract infections [6], central nervous system infections like meningitis [7], and hepatobiliary disorders, especially in immunocompromised individuals and transplant recipients [8], are among the human infections caused by B. hinzii, despite its usual association with rodents and birds. Though B. pertussis is a well-known Bordetella species associated with respiratory illness, the pathogenic role of B. hinzii in humans is still being explored. Only 18 human infection cases have been documented worldwide till 2026, with the first Asian case (pneumonia) occurring in China in 2021 [3,5]. 

The identification of this bacteria in clinical settings is challenging due to its phenotypic similarity to other Bordetella species. Conventional biochemical methods often fail to differentiate it, necessitating molecular diagnostics like 16S rRNA sequencing or matrix-assisted laser desorption/ionization-time of flight (MALDI-TOF) mass spectrometry for accurate detection [5-7]. Occupational and environmental exposure to its zoonotic reservoirs, poultry and rodents, pose potential risks [2]. Also, nosocomial transmission has been suspected in immunocompromised individuals without direct animal contact [9,10].

Pregnancy itself induces a physiological immunocompromised state, and this vulnerability is further exacerbated by conditions like sickle cell anemia (SCA), through chronic hemolysis, functional asplenia, and recurring vaso-occlusive crises. Infants born prematurely to mothers with SCA are at higher risk of infection and adverse outcomes.

We report a rare case of antenatal sepsis caused by B. hinzii in a pregnant woman with sickle cell disease, complicated by intrauterine fetal demise. This case expands the known clinical spectrum of B. hinzii infection and highlights the importance of considering opportunistic and atypical pathogens in high-risk obstetric patients.

## Case presentation

A 27-year-old female patient, a known case of homozygous sickle cell disease (HbSS pattern), was admitted to a tertiary care hospital at 24 weeks of gestation (G2P1A1) with complaints of fever for five days, swelling over the feet for four days, breathlessness for two days, and decreased fetal movements for one day. No recent history of burning micturition and symptoms of GIT infections were present. Patient visited a relative's house where she was exposed to chickens a few days before presenting to the health care facility.

On general physical examination, the patient was conscious and oriented, had tachycardia (108/min), slightly raised blood pressure (140/90 mm of Hg), pallor and oedema of the feet were present. Patient was febrile, tachypneic but no documented evidence of hypoxia was present. Abdominal examination revealed 24 weeks of gestational height with hepatosplenomegaly and decreased fetal heart sound.

Her routine investigations revealed severe anemia (Hb 5.7 g/dl) (reference range: 11.0-14.0 g/dL), leukocytosis (total leukocyte count (TLC) 18,600/mm³) (reference range: 4,000-11,000/mm³), and raised C-reactive protein (22 mg/L) (reference range: <5 mg/L). Renal function tests were within normal limits, including serum creatinine of 0.6 mg/dL (reference range: 0.5-1.1 mg/dL). Liver function tests were also normal, with total bilirubin 0.9 mg/dL (reference range: 0.2-1.2 mg/dL), aspartate aminotransferase (AST) 22 U/L (reference range: 10-40 U/L), and alanine aminotransferase (ALT) 16 U/L (reference range: 7-56 U/L). Chest X-ray revealed bilateral patchy infiltrates consistent with lower respiratory tract infection. Ultrasonography of the abdomen and pelvis which was done immediately after admission showed a single intrauterine fetus of 24 weeks with absent cardiac activity, anhydramnios, and hepatosplenomegaly. ECG was normal, and 2D-ECHO showed mild concentric left ventricular hypertrophy with preserved ejection fraction. Original radiological images could not be obtained because of institutional archival constraints.

With the provisional diagnosis of antenatal case (ANC) (G2P1A1), known case of homozygous sickle cell disease (HbSS pattern) with anhydramnios with severe anemia and fetal demise, a blood sample of the patient was sent for blood culture. She remained hemodynamically stable and did not require intensive care unit admission. The patient was managed empirically with intravenous meropenem (1 gm twice daily), vancomycin (1 gm twice daily) and levofloxacin (500 mg once daily on alternate days) and other supportive treatment like blood transfusion support, antihypertensive treatment, and close maternal monitoring. Following treatment, gradual clinical improvement was observed with stabilization of general condition and resolution of sepsis-related symptoms. The total duration of hospital stay was approximately 10 days. Although maternal recovery was achieved, the pregnancy outcome was complicated by intrauterine fetal demise.

One blood culture set was obtained before the start of antibiotics, conventional blood culture was performed, and subculture was done on blood agar, chocolate agar, and MacConkey agar. After 48 hours of incubation at 37 C in 5% CO2 on blood agar, the colonies were small (1-2 mm), circular, convex, and translucent with a smooth, glistening surface and regular margins. The growth was greyish, non-hemolytic, and easily emulsifiable. On MacConkey agar, colonies were non-lactose fermenting. Microscopy revealed motile, Gram-negative coccobacilli that tested positive for both catalase and oxidase. On biochemical testing, the isolate utilized citrate, but did not ferment glucose, lactose, or sucrose, and tested negative for indole, methyl red, and urease.

The pure isolated colonies were further processed by the VITEK2 automated system (bioMérieux, Marcy-l'Étoile, France) for identification and antimicrobial susceptibility testing. The strain was confirmed as Bordetella hinzii (excellent identification).

Antimicrobial susceptibility done by Kirby-Bauer disk diffusion and minimum inhibitory concentration testing by VITEK2 showed similar results with susceptibility to ceftazidime, cefepime, piperacillin/tazobactam, meropenem, co-trimoxazole, and levofloxacin. The isolate was resistant to ampicillin, cefuroxime, cefoxitin, ceftriaxone, aztreonam, imipenem, amikacin, tobramycin and gentamicin. Interpretation was performed using non-Enterobacterales breakpoints as per CLSI 35th edition [11]. No standardized guidelines exist specifically for B. hinzii. Repeat blood culture after initiation of therapy was sterile.

**Figure 1:**
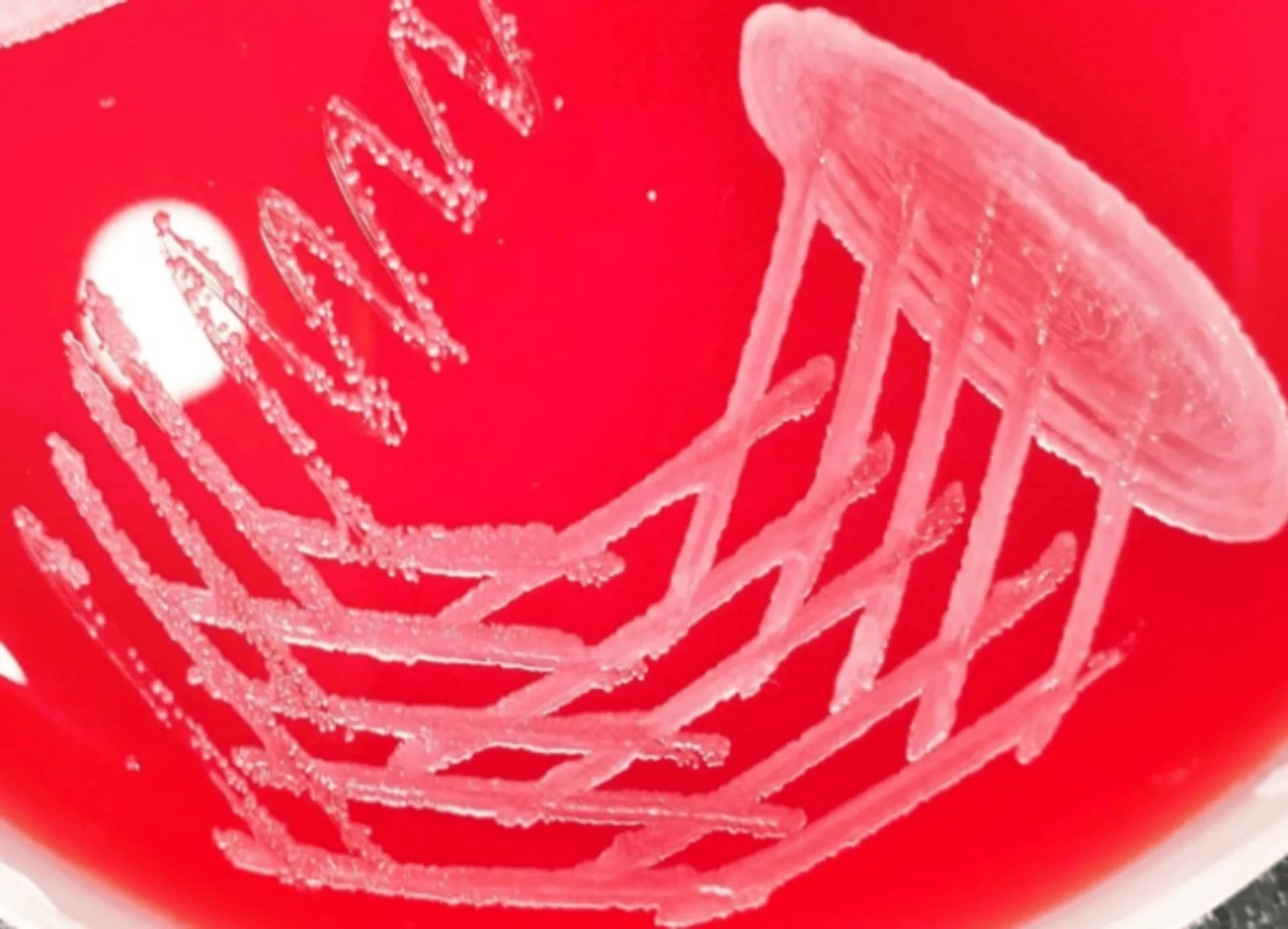
B. hinzii colonies on blood agar

 Colony morphology on blood agar is shown in Figure 1.

## Discussion

First identified as an avian respiratory tract isolate in the 1990s, B. hinzii has since been recognized in human infections, particularly in individuals with compromised immune systems [2].

Chen et al. reported the first Asian case of B. hinzii infection from China, with no prior documented cases from India at that time [5]. However, the current case report of antenatal sepsis caused by B. hinzii in a pregnant woman with SCA represents the first case from India and probably the first global report of B. hinzii antenatal sepsis with intrauterine fetal demise to the best of authors’ knowledge, contributing to the spectrum of clinical presentations. Pregnancy-associated immune modulation combined with functional asplenia and immune dysfunction in sickle cell disease may have predisposed to invasive opportunistic infections.

Published cases of B. hinzii infection predominantly involve adult males, particularly with immunocompromising conditions.

Fabre et al. reported a 52-year-old male with pulmonary infection, while Maison-Fomotar et al. described pneumonia and bacteremia in a 49-year-old male with SARS-CoV-2 [3,4]. Other cases include a 48-year-old liver transplant recipient with cholangitis [8], a 55-year-old male with a cervical abscess [12], and meningitis in a 69-year-old kidney transplant recipient [7]. Female cases are less frequent but include a urinary tract infection [6] and a pulmonary infection following avian exposure [4]. Pediatric colonization has also been observed in cystic fibrosis patients [13]. The present case is also a 27-year-old female patient with SCA in antenatal sepsis.

Various published reports suggested a zoonotic origin of B. hinzii, often linked to avian or poultry exposure, as referred here subsequently. Vandamme et al. first isolated B. hinzii from the respiratory tracts of poultry and poultry handlers [2]. Fabre et al. documented pulmonary infection in a woman with pet parrot exposure [4]. Similarly, Jiyipong et al. identified these environmental reservoirs by detecting B. hinzii from poultry and rodents in Southeast Asia [9]. In the current case also poultry exposure was reported by the patient and the presence of underlying sickle cell disease could have facilitated the infection. All these findings support the hypothesis of B. hinzii as a potentially zoonotic pathogen, particularly in immunocompromised hosts. However, several infections have been reported in the absence of avian exposure. Other cases by Arvand et al. and Negishi et al. suggest alternative routes, such as nosocomial or environmental transmission [8,12].

B. hinzii infections display a broad clinical spectrum, primarily affecting immunocompromised individuals. Reported cases include pulmonary infections in patients with avian exposure, pneumonia and bacteremia in a SARS-CoV-2-infected patient and chronic cholangitis in a liver transplant recipient [4,3,8]. Other presentations include a cervical abscess [12], meningitis in a kidney transplant patient [7], and pneumonia in an immunocompromised man in China [5]. Spilker et al. reported colonization in cystic fibrosis patients [13]. The present case of a 27-year-old pregnant woman with SCA presenting with antenatal sepsis and intrauterine fetal demise is unique and expands the known manifestations of B. hinzii infection. Maternal systemic inflammatory response and placental hypoperfusion secondary to sepsis may have contributed to intrauterine fetal demise.

The antimicrobial susceptibility profiles of B. hinzii demonstrate variability, emphasizing the importance of culture-guided therapy. In the present case, the isolate was susceptible to ceftazidime, cefepime, piperacillin/tazobactam, meropenem, levofloxacin and co-trimoxazole and resistant to ampicillin, ceftriaxone, gentamicin, aztreonam, imipenem, and amikacin. Fabre et al. reported susceptibility to piperacillin/tazobactam and ciprofloxacin, though resistance to ampicillin was noted [5]. Arvand et al. also found susceptibility to piperacillin/tazobactam and cefotaxime in a liver transplant patient, again with ampicillin resistance [8]. Maison-Fomotar et al. noted meropenem susceptibility [3]. Collercandy et al. observed resistance to aminoglycosides and third-generation cephalosporins, but susceptibility to meropenem and cefepime [6]. Pechacek et al. reported a CNS infection with resistance to aminoglycosides and first-generation cephalosporins [7]. Spilker et al. emphasized variable patterns and the importance of distinguishing between colonization and infection [13].

B. hinzii infections have demonstrated various outcomes. Maison-Fomotar et al. described a patient with SARS-CoV-2 co-infection who developed pneumonia and bacteremia; although the patient survived, intensive care was required [3]. Pechacek et al. reported meningitis in a kidney transplant recipient; while the outcome was not specified but such CNS infections in immunosuppressed patients are typically associated with poor prognosis [7]. Spilker et al. noted respiratory colonization in cystic fibrosis patients without progression to overt disease [13]. All these cases highlight B. hinzii’s potential for causing invasive disease and emphasize the increased risk of complications in vulnerable patient populations. All the various published cases have been summarized in Table 1.

**Table 1:** Previous documented cases of B. hinzii AIDS = acquired immunodeficiency syndrome; API = Analytical Profile Index; NFT = non-fermenting; NE = non-enteric; ANC = antenatal care; DNA = deoxyribonucleic acid; DNA-rRNA = DNA-ribosomal ribonucleic acid; MALDI-TOF MS = matrix-assisted laser desorption/ionization time-of-flight mass spectrometry; NR = not reported; PAGE = polyacrylamide gel electrophoresis; rRNA = ribosomal ribonucleic acid; VITEK 2 = VITEK 2 automated identification system (bioMérieux, Marcy-l'Étoile, France); NA = not available

Reference	Year	Clinical diagnosis	Age/ gender	Epidemiology	Immunosuppression status	Method of identification	Antibiotics	Outcome
Vandamme P, et al. [2]	1995, France	NR	NR	NR	NR	Identification of new species through PAGE of whole-cell proteins, fatty acid methyl ester analysis, DNA-DNA and DNA-rRNA hybridization	NR	NR
Funke G, et al. [14]	1996, Switzerland	Pulmonary symptoms	51/M	NR	none	API 20 NFT (NE) system, alkali production from malonate and PAGE of whole-cell proteins	Amoxicillin-clavulanic acid, ciprofloxacin	Recovered
Cookson BT, et al. [15]	1994, USA	Bacteremia	42/M	NR	Immunosuppressed (AIDS)	API NFT, whole-cell fatty acid analysis and DNA-DNA hybridization	Vancomycin; ceftriaxone; ceftriaxone, rifampin	Recovered
									Kattar MM, et al. [16]	1999, USA	Cholestasis and bacteremia	69/M	Had attended a cookout at a farm 2 weeks before admission	None	Traditional biochemical testing and 16S rRNA gene sequence analysis	Ampicillin-sulbactam, cefotetan; ampicillin, gentamicin, metronidazole; ticarcillin- sulbactam, ciprofloxacin	Died
																		Gadea I, et al. [17]	2000, Spain	Respiratory tract infection	NR	No avian exposure	Immunosuppressed (AIDS)	Traditional biochemical testing and 16S rRNA gene sequence analysis	NR	NR
Arvand M, et al. [8]	2001, Germany	Chronic cholangitis	29/M	NR	Immunosuppressed (liver transplant recipient)	Traditional biochemical testing and 16S rRNA gene sequence analysis	Piperacillin-tazobactam, gentamicin; amphotericin B, flucytosine, vancomycin, and meropenem	Died									
									Fry NK, et al. [18]	2007, United Kingdom	Flu-like symptoms	79/M	NR	Immunosuppressed (myelodysplastic syndrome)	Genotypic methods and gene sequence analysis (ompA, 16S rRNA gene)	Amoxicillin, clavulanic acid; vancomycin, ceftazidime	Recovered
																		Hristov AC, et al. [19]	2008, USA	Fevers, full-body rash, fatigue; respiratory distress	36/F	NR	Immunosuppressed (Epstein–Barr virus viremia and lymphoma)	Cellular fatty acid analysis and 16S rRNA gene sequence analysis	Amoxicillin-clavulanic acid, oxacillin, vancomycin, trimethoprim– sulfamethoxazole, doxycycline, linezolid, meropenem, itraconazole.	Died
																											Palacián Ruiz MP, et al. [20]	2013, Spain	Respiratory symptoms	85/F	NR	NR	MALDI-TOF-MS; 16S rRNA gene sequence analysis	Amoxicillin-clavulanate	Unclear
									Fabre A, et al. [4]	2013, France	Pulmonary infection	43/M	Avian exposure	Immunosuppressed (leukemia, diabetes, vascular hypertension, and non-symptomatic chronic bronchiectasis before the allograft)	MALDI-TOF-MS; 16S rRNA gene sequence analysis	Ciprofloxacin; trimethoprim/ sulfamethoxazole; piperacillin/tazobactam, ciprofloxacin; vancomycin	Recovered									
Fabre A, et al. [4]	2014, France	Chronic obstructive pulmonary disease	74/M	No recent exposure to pets and poultry	Immunosuppressed (vascular hypertension, dyslipidemia, prostate cancer, ischemic heart disease)	MALDI-TOF-MS; 16S rRNA gene sequence analysis	Piperacillin/tazobactam, vancomycin																			
								Recovered																		
								María González M, et al. [21]										2019, Spain	Infective endocarditis	79/M	No contact with poultry	Cirrhosis, kidney disease and diabetes	MALDI-TOF-MS; 16S rRNA gene sequence analysis	Cefotaxime, imipenem, levofloxacin, erythromycin, minocycline, and co-trimoxazole, meropenem, piperacillin-tazobactam	Unclear
Zohourian H, et al. [22]	2019, USA	Bacteremia and infective endocarditis	53/F	No avian exposure	Ulcerative colitis		Ceftazidime																			Recovered
Negishi T, et al. [12]	2019, Japan	Soft tissue abscess	63/M	none	none	16S rRNA gene sequencing	Sitafloxacin																			Recovered
Kampmeier S, et al. [23]	2020, Germany	Pancreatic abscess	42/M	none	Fatty liver disease	16S rRNA gene sequencing	Tigecycline	Recovered									
Chen D, et al. [5]	2021, China	Pneumonia	67/F	No avian exposure	Cerebral hemorrhage	MALDI-TOF-MS; 16S rRNA gene sequence analysis	Cefmetazole	Recovered
									Sharma S, et al. [24]	USA	Respiratory tract infection	45/M	No avian exposure	none		Piperacillin/tazobactam	Recovered
																		Wang L, et al. [25]	2025, China	Pneumonia	68/M	NR	chronic obstructive pulmonary disease	MALDI-TOF MS	Meropenem, levofloxacin, doxycycline, trimethoprim-sulfamethoxazole, minocycline, ciprofloxacin, moxifloxacin, tigecycline	Recovered
Zhou J, et al. [26]	2025, China	Postoperative pulmonary infection	50/M	NR	none	16S rRNA gene sequencing	Piperacillin/sulbactam combined with levofloxacin	Recovered																		
									Kulkarni M, et al. (this study)	2026, India	ANC	27/F	Avian exposure	none	VITEK 2	Ceftazidime, cefepime, piperacillin/tazobactam, meropenem and co-trimoxazole, and levofloxacin	Recovered									

The present case developed antenatal sepsis and survived, but the fetus succumbed to intrauterine death, reflecting adverse maternal-fetal consequences.

Limitations

Placental evaluation and molecular confirmation by 16S rRNA sequencing or MALDI-TOF could not be performed due to the resource-limited setting.

## Conclusions

We report the first case (to the best of the authors' knowledge) of B. hinzii sepsis from a 24-week antenatal patient with sickle cell disease “SS pattern” with intrauterine fetal demise having exposure to poultry.

The difficulties in suspecting and identifying B. hinzii infections highlights the need for more studies to clarify the epidemiology, pathophysiology, and management of this rarely isolated zoonotic pathogen. With a limited number of documented cases and the wide range of clinical presentations, research targeting multisectoral “One Health Approach” could provide valuable information. This case also highlights the importance of automated bacterial identification systems to better patient management and epidemiological purposes.
